# Continuous Remote Patient Monitoring Shows Early Cardiovascular Changes in COVID-19 Patients

**DOI:** 10.3390/jcm10184218

**Published:** 2021-09-17

**Authors:** Arik Eisenkraft, Yasmin Maor, Keren Constantini, Nir Goldstein, Dean Nachman, Ran Levy, Michael Halberthal, Netanel A. Horowitz, Ron Golan, Elli Rosenberg, Eitan Lavon, Ornit Cohen, Guy Shapira, Noam Shomron, Arik Ben Ishay, Efrat Sand, Roei Merin, Meir Fons, Romi Littman, Yftach Gepner

**Affiliations:** 1 Institute for Research in Military Medicine, The Hebrew University Faculty of Medicine, P.O. Box 12272, Jerusalem 9112102, Israel; deannahman@gmail.com; 2 The Israel Defense Force Medical Corps, P.O. Box 12272, Jerusalem 9112102, Israel; 3 Biobeat Technologies Ltd., 22 Efal St., Petah Tikva 4951122, Israel; arik@bio-beat.com (A.B.I.); efratsand@gmail.com (E.S.); m.roei@bio-beat.cloud (R.M.); meir@bio-beat.com (M.F.); romi@bio-beat.cloud (R.L.); 4 Wolfson Medical Center, 62 Ha-Lokhamim St. 62, Holon 58100, Israel; yasminm@wmc.gov.il (Y.M.); ORNITC@wmc.gov.il (O.C.); 5 The Sackler Faculty of Medicine, Tel Aviv University, P.O. Box 39040, Tel Aviv 6997801, Israel; guyspersonal@gmail.com (G.S.); nshomron@post.tau.ac.ilethic (N.S.); 6 Department of Epidemiology and Preventive Medicine, School of Public Health, Sackler Faculty of Medicine and Sylvan Adams Sports Institute, Tel Aviv University, P.O. Box 39040, Tel Aviv 6997801, Israel; kconstantini@mail.tau.ac.il (K.C.); goldsnir@tauex.tau.ac.il (N.G.); gepner@tauex.tau.ac.il (Y.G.); 7 Heart Institute, Hadassah Ein Kerem Medical Center, P.O. Box 911201, Jerusalem 9112102, Israel; 8 Maccabi Healthcare Services, P.O. Box 50493, Tel Aviv 68125, Israel; levy_ra@mac.org.il; 9 General Directorate Rambam Health Care Campus, P.O. Box 9602, Haifa 3109601, Israel; m_halberthal@rambam.health.gov.il (M.H.); n_horowitz@rambam.health.gov.il (N.A.H.); 10 The Bruce Rappaport Faculty of Medicine, Technion, P.O. Box 9649, Haifa 3525433, Israel; 11 The Baruch Padeh Medical Center Poriya, The Faculty of Medicine in Galilee, Bar Ilan University, Upper Galilee, Poria 1528001, Israel; RoGolan@poria.health.gov.il; 12 Internal Medicine A, The Soroka University Medical Center, Ben-Gurion University of the Negev, P.O. Box 151, Be’er Sheva 84101, Israel; elliro@clalit.org.il; 13 The Kaplan Medical Center, The Hebrew University Faculty of Medicine, P.O. Box 1, Rehovot 76100, Israel; eitan@clalit.org.il; 14 Faculty of Health Science, Ben-Gurion University of the Negev, P.O. Box 653, Be’er Sheva 8410501, Israel

**Keywords:** COVID-19, cardiopulmonary parameters, high-risk population, remote patient monitoring, artificial intelligence

## Abstract

COVID-19 exerts deleterious cardiopulmonary effects, leading to a worse prognosis in the most affected. This retrospective multi-center observational cohort study aimed to analyze the trajectories of key vitals amongst hospitalized COVID-19 patients using a chest-patch wearable providing continuous remote patient monitoring of numerous vital signs. The study was conducted in five COVID-19 isolation units. A total of 492 COVID-19 patients were included in the final analysis. Physiological parameters were measured every 15 min. More than 3 million measurements were collected including heart rate, systolic and diastolic blood pressure, cardiac output, cardiac index, systemic vascular resistance, respiratory rate, blood oxygen saturation, and body temperature. Cardiovascular deterioration appeared early after admission and in parallel with changes in the respiratory parameters, showing a significant difference in trajectories within sub-populations at high risk. Early detection of cardiovascular deterioration of COVID-19 patients is achievable when using frequent remote patient monitoring.

## 1. Introduction

Coronavirus disease 2019 (COVID-19) is a multi-system disease with a wide range of clinical manifestations, from asymptomatic patients to a simple influenza-like illness, or to a fulminant disease comprised of acute respiratory distress syndrome (ARDS) and pulmonary insufficiency [1]. As the understanding of the disease evolved, it was shown that COVID-19 damages epithelial and endothelial cells in numerous tissues leading to SARS-CoV-2 (severe acute respiratory syndrome coronavirus 2)-related multi-organ failure [1,2,3,4]. Previous studies have demonstrated substantial damage to the cardiovascular system with systemic hemodynamic and direct cardiac effects, a negative prognostic sign leading to increased morbidity in individuals with underlying cardiovascular diseases [1,5,6]. Respiratory parameters such as respiratory rate (RR) and blood oxygen saturation (SpO_2_) are key measurements in the assessment and prognosis of COVID-19 patients [7]. So far, these were carried out by infrequent spot measurements, mainly due to the capabilities of currently used devices.

Novel technologies are leading us to the next generation of care provision, as they broaden the capacity to remotely monitor patients, providing optimized support and protection both for COVID-19 patients and for health care providers by reducing direct contact without compromising the treatment given to isolated patients, as well as lightening the strain on the medical teams and providing early detection of patient deterioration [8,9,10,11,12,13]. Moreover, frequent monitoring of multiple vital physiological parameters may allow better care for patients who are acutely ill, for hospital-at-home of stable patients or those in imminent danger, and early discharge of admitted patients [5,9]. It is also accepted that such a system would be of benefit if it frequently and automatically measures numerous vital signs, improves patient surveillance, and, ultimately, improves patient outcomes [8,14,15,16]. Frequent remote patient monitoring (RPM) systems are potentially better equipped to detect and alert of changes since the vital sign measurements are taken continuously and for longer periods [8,9]. Combining advanced wearable devices with machine learning in various clinical settings may allow reliable detection of changes in population health status, including follow-up of COVID-19 patients and at an earlier stage, helping to control the spread of the pandemic [9,17,18].

Several reports so far have identified differences in COVID-19 infection rates, symptom severity, and mortality between sex, age, and body mass index (BMI) categories [19,20,21,22]; however, whether the physiological response of key cardiovascular and respiratory parameters during the course of hospitalization is different between individuals remains unknown. This knowledge may help in further understanding the clinical course of the disease and preventing clinical deterioration, with highlighted importance due to the rapid deterioration often seen in COVID-19 patients [1].

This retrospective observational multi-center study aimed to determine the trajectory of nine physiological parameters amongst COVID-19 patients admitted to isolation units in Israeli medical centers. A special emphasis was given to characterizing the disease progression among these patients, and to the identification of differences in physiological responses over time of sub-groups according to age, sex, and BMI.

## 2. Materials and Methods

### 2.1. Study Design

**Figure 1:**
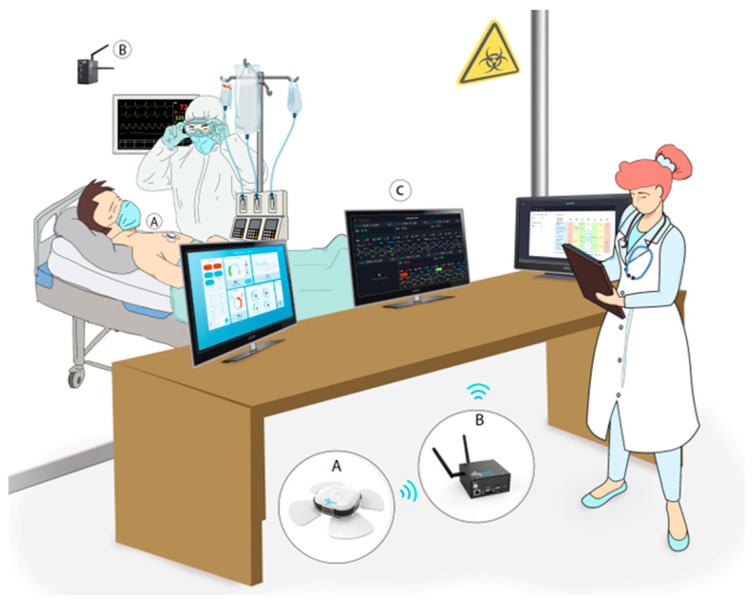
The remote patient monitoring (RPM) system is deployed in isolation units. The chest-patch sensor was attached to all moderate to severe COVID-19 patients included in the study. This RPM system provides automatic measurement of 15 vital signs every 5 to 15 min. Data is transmitted through Bluetooth low energy (BLE) from the sensors (**A**) to gateways (**B**) installed in the isolation units and from there through Wi-Fi or a SIM card to a cloud-based medical management application available to health care providers on any web platform (**C**), allowing to monitor all admitted patients at once. The monitor shows the vital signs in real-time, provides alerts and has an integrated early warning score system.

This retrospective observational and non-interventional multi-center study was conducted between 3 March 2020, and 22 May 2020. We included COVID-19 patients 18 years and older that were admitted into five COVID-19 dedicated isolation units in Israel, and continuously monitored using non-invasive, wearable, and wireless photoplethysmography (PPG)-based chest-monitors (BB-613WP, Biobeat Technologies Ltd., Petah Tikva, Israel). The monitors were attached and activated upon admission, with measurements automatically taken every 15 min and transmitted in real-time to a cloud-based web application used by the medical staff (Figure 1). Big data analysis was conducted using advanced AI and bioinformatics tools.

Designated gateways (BB-SGWB Smart Gateway Box/BB-90000300100, Biobeat Technologies Ltd., Petah Tikva, Israel) were installed in the isolation units to ensure continuous monitoring and data transmission of all measurements (Figure 1). Physiological parameters included in the analysis were heart rate (HR), SpO_2_, RR, cuff-less non-invasive blood pressure (BP), stroke volume (SV), cardiac output (CO), cardiac index (CI), systemic vascular resistance (SVR), and body temperature.

### 2.2. Inclusion and Exclusion Criteria

Inclusion criteria were male and female adults above the age of 18 years, with a positive COVID-19 PCR test, admitted to designated COVID-19 isolation units in a moderate to a severe condition as defined by the local health care providers, but with no need for admission in an intensive care unit (ICU). Exclusion criteria were pregnancy, the need for ventilation, and admission to an ICU.

### 2.3. The Remote Patient Monitoring (RPM) System

The FDA-cleared chest-monitor device used in this study (Figure 1) utilizes a unique reflective PPG technology, in which specific wavelengths of light are transmitted onto the skin, and the reflected light is collected by a photodiode detector positioned near the light source transmitter. The sensor tracks vital signs derived from changes in the pulse contour, following a simple offset baseline trimonthly calibration process using an approved non-invasive, cuff-based device, and is based on Pulse Wave Transit Time (PWTT) technology combined with Pulse Wave Analysis (PWA) (for error% levels please see validation results in [23,24,25,26]). Patients were monitored throughout hospitalization, and sensors were replaced if hospitalization lasted longer than the 6-day battery life. The same sensors were not used to monitor different patients.

### 2.4. Ethical Approval

All methods were carried out under relevant guidelines and regulations and approved by the institutional review boards as follows: approval 0077-20-WOMC was provided by the Wolfson Medical Center’s IRB, approval 0048-20-POR was provided by the Padeh Poriya Medical Center’s IRB, approval 0506-20-RMB was provided by the Rambam Medical Center’s IRB, approval 0193-20-KMC was provided by the Kaplan Medical Center’s IRB, and approval 0421-20-SOR was provided by the Soroka Medical Center’s IRB. Informed consent was waived by all IRBs.

### 2.5. Statistical Analysis

Global outliers were picked using PCA and histogram examination. On a per-patient level, outlier observations with a difference from the mean that was greater than Q3 + 3xIQR were discarded. Differences between the studied groups were determined using an independent t-test when the data satisfied test requirements. Equal variances were assumed if Barlett and Levene’s tests came out significant. Wilcox test was performed for metrics with non-normal distribution. For key physiological variables recorded during the first 2 h of admission, normality was assessed using the Shapiro test QQ-plots, allowing the removal of extreme outliers. Then, differences between groups were determined using repeated-measures ANOVA. Where a significant main effect was found, a Tukey’s post hoc test was performed. The data were fitted to a linear mixed model with nlme 3.1, with sex, age range, and BMI as coefficients, then tested using ANOVA. Pairwise Wilcox was used for post hoc testing when the sample sizes were sufficiently large (n > 50). Trend estimation figures with less than 1000 observations were performed using LOESS, while larger trend data was performed using GAM. All other results are presented as means ± SD. Statistical analyses were considered significant if *p* < 0.05. All statistical analyses were made using R version 3.6.3 (GBIF.org, Copenhagen, Denmark) [27].

## 3. Results

**Figure 2:**
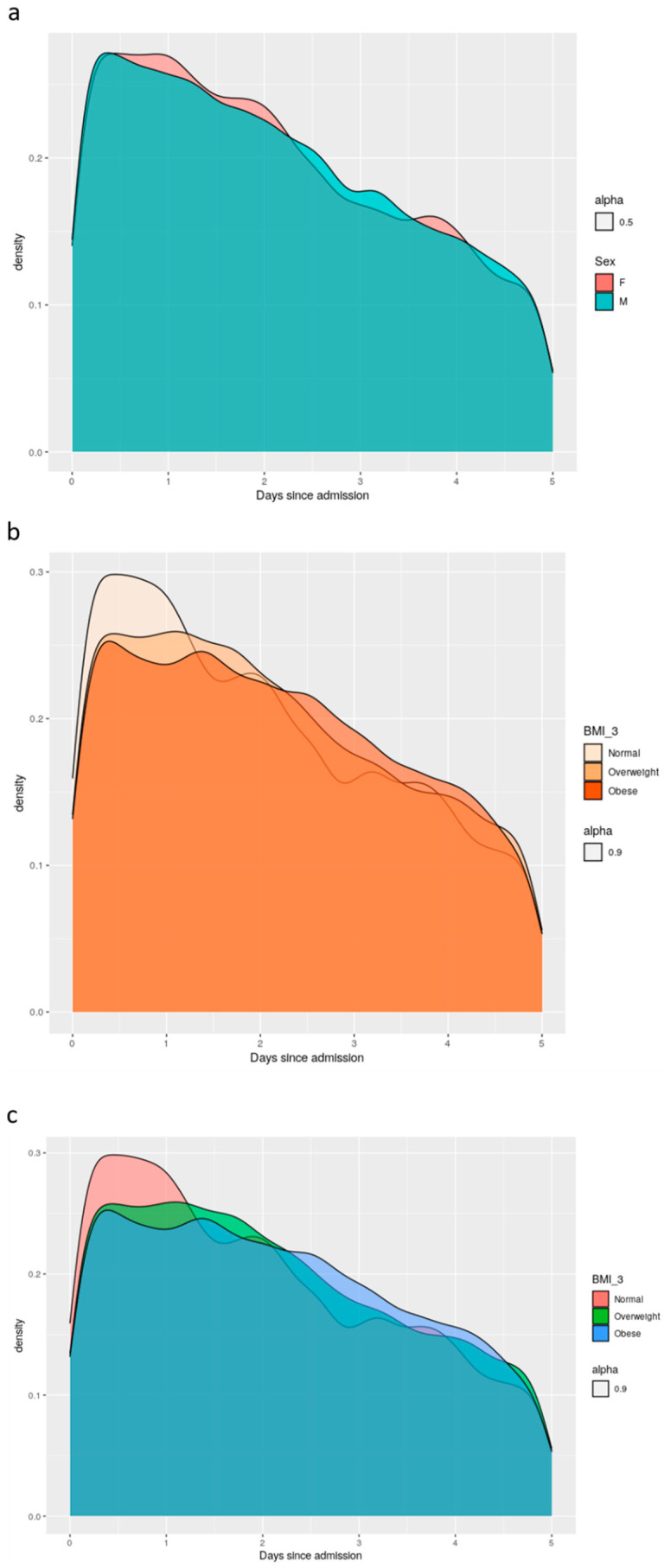
The number of subjects per group throughout the entire monitoring period. (**a**) division by sex; (**b**) division by age groups (young: 18–40 years; middle age: 40–60 years; elderly: 60–80 years; >80 years); (**c**) division by body mass index (BMI; normal weight: <24.9 kg∙min^−2^; overweight: 25–29.9 kg∙min^−2^; obese: >30 kg∙min^−1^). The breakdown of patients’ numbers by day was 492 at 24 h, 408 at 48 h, 294 at 72 h, 204 at 86 h, 130 at 120 h, 92 at 144 h, 66 at 168 h, 49 at 192 h, 43 at 216 h, 33 at 240 h, and 21 at 264 h.

Initially, 571 patients participated in the study. Subjects with less than 24 h of continuous tracking data were excluded, with 492 patients and a total of 3,215,334 measurements remaining. As can be seen in Figure 2a–c, the number of patients decreased throughout the monitoring period due to patients’ discharge from the hospital, transfer to ICUs, or death. As a result, we focused our final analysis on the first five days (120 h) after admission. The raw, unfiltered data included measurements collected during an average monitoring period of 75.26 h (range 0–455), with 245.67 ± 226.39 observations (±standard deviation) per patient. The analyzed filtered data included patients with at least 24 h of tracking and included observations from the first five days only. Measurements were collected during an average monitoring period of 74.78 h (range 24–120) per patient (174.89 ± 110.58).

Mean values during the first 2 h of monitoring (average of 8 measurements on admission) served as a baseline monitoring period for each subject. For the entire study population, baseline measurements were 82 ± 10 for HR, 95 ± 2 for SpO_2_, 129 ± 17 for SBP, 74 ± 11 for DBP, 70 ± 12 for SV, 5.7 ± 1.3 for CO, 1368 ± 296 for SVR, and 35.7 ± 3.6 for body temperature.

Subject characteristics, along with mean values during the baseline monitoring period for each subject for body temperature, SpO_2_, RR, HR, systolic BP (SBP), diastolic BP (DBP), CO, CI, and SVR for each sub-group are presented in Table 1.

For baseline physiological measurements, an ANOVA analysis revealed a significant (*p* < 0.01) difference between men and women for body temperature, SpO_2_, HR, DBP, and CO. When analyzing age groups, significant (*p* < 0.003) interactions were seen for all physiological measures recorded during the first 2 h of admission. Lastly, only SpO_2_, RR, CO, and CI were significantly (*p* < 0.01) different between BMI groups upon admission.

**Figure 3:**
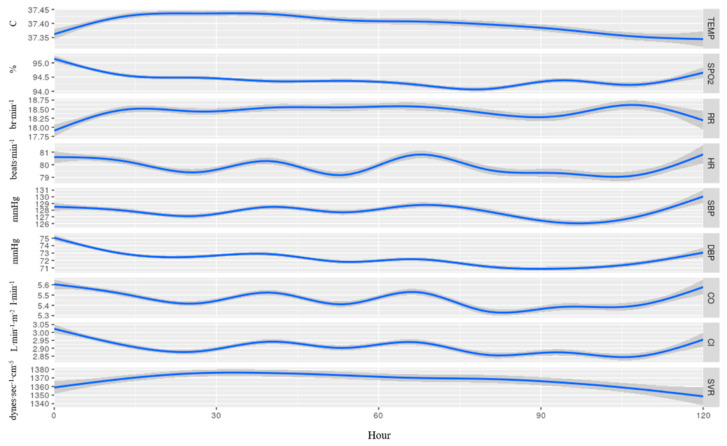
Average recorded measurements among 130 patients that were continuously monitored for 5 days from admission, without separation to sub-groups. Temp, temperature; SpO_2_, blood oxygen saturation; RR, respiratory rate; HR, heart rate; SBP, systolic blood pressure; DBP, diastolic blood pressure; CO, cardiac output; CI, cardiac index; SVR, systemic vascular resistance. The blue line represents the mean value of each vital and the 95% confidence interval appears in gray.

Figure 3 provides an overall description of the nine vital signs in the 130 patients that fully completed five days of continuous monitoring. Overall, within the first 24 h, we found a significant increase in temperature, RR, and SVR, and a significant decrease in SpO_2_, DBP, CO, and CI (*p* < 0.01 for all). These changes all appeared at the same time. For HR, SBP, CO, and CI, the changes appeared in a repetitive pattern.

**Figure 4:**
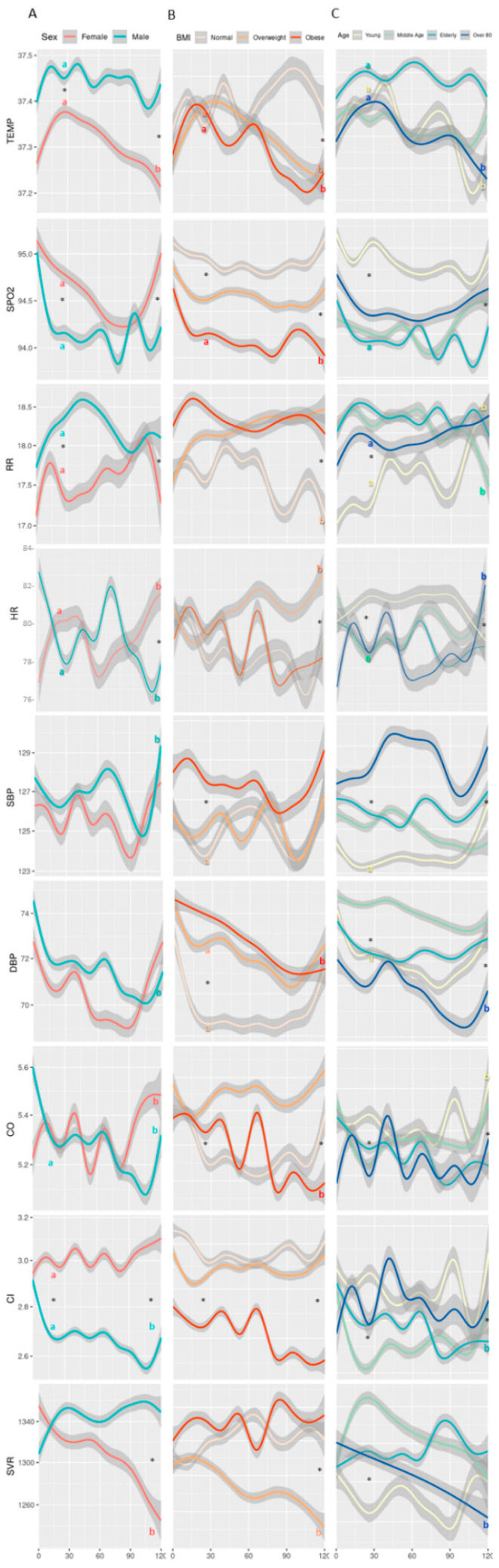
Measured vital signs by (**A**) sex, (**B**) BMI, and (**C**) age group, over the course of the first 5 days of tracking. Definitions of BMI and age groups are provided in Figure 2. Temp, temperature; SpO_2_, blood oxygen saturation; RR, respiratory rate; HR, heart rate; SBP, systolic blood pressure; DBP, diastolic blood pressure; CO, cardiac output; CI, cardiac index; SVR, systemic vascular resistance. Each line represents mean values of the vitals, and the 95% confidence interval appears in gray. * Significant (*p* < 0.05) difference between groups for a given time-point; ^a^ significant (*p* < 0.05) difference between 24 h and baseline within a group; ^b^ significant (*p* < 0.05) difference between 120 h and baseline within a group.

Further analysis is provided in Figure 4, where we show the results of repeated-measures ANOVA tests performed to determine differences between groups during the same timeframe. In both males and females, the temperature increased during the first 24 h of monitoring, reaching significantly higher values among males (*p* < 0.001). From the second day, females showed a significant decrease in temperature until the fifth day (*p* < 0.001). In the age group, temperature among the elderly was higher (*p* < 0.001) during the whole 5 days.

SpO_2_ decreased in both males and females during the first 48 h (*p* < 0.001 for both), with a higher decrease among males. Starting from 72 h since admission, and throughout the next two days, females showed a quicker return to the baseline levels, while males remained with lower values (*p* < 0.03 between sex). In the age group, the young maintained SpO_2_ levels throughout the five days of hospitalization with a slight reduction (*p* = 0.044), while other sub-groups showed dynamic changes with lower SpO_2_ values.

Within the first 48 h from admission, RR increased significantly in males, obese patients, and in all age sub-groups (*p* < 0.01) except the young. Throughout the whole five days of hospitalization, the overweight and obese sub-groups had higher RR as compared to the normal weight sub-group (*p* < 0.01). The young showed a significant increase (*p* = 0.044) in RR.

During the first 48 h, HR dropped among men, while it increased among women (*p* < 0.01 in both). From then on, the dynamics of HR behaved in opposite manners between sex, until day 5, in which both showed an increase, with females showing a significant increase from baseline (*p* < 0.01).

Changes in SBP over five days of hospitalization were significantly different between age groups (*p* = 0.002). Both sexes showed a decrease in DBP values during the first 4 days, women to a higher extent than men (*p* < 0.001), and in the fifth day both showed an increase with values among women returning to the baseline level. Patients over 80 years showed relatively lower DBP values during the 5 days, reaching a nadir at day 4 (*p* < 0.001). The young and elderly showed a decreasing trend in the first 3 days (*p* < 0.01), returning close to baseline levels by day 5. Normal weight patients had a sharp decrease during the first 24 h (*p* < 0.001), maintaining this level until the 4th day, followed by a sharp increase during the 5th day to baseline levels. Both overweight and obese had a milder decrease during the first 4 days with overweight returning to baseline levels on the 5th day, and the obese returning to a lower level than the baseline (*p* = 0.02).

Males had a sharp decrease in CO in the first 24 h (*p* < 0.01), kept stable over the next 48 h, followed by a sharp decrease in the 4th day, and an increase in the 5th day to a level below the baseline. Females showed dynamic changes reaching peak high levels on each of days one to three, followed by a drop shortly after every peak, followed by a constant increase until reaching the highest peak on day 5 (*p* < 0.01). Patients over 80 years had a dynamic pattern of changes with peaks at 24 h and 48 h from admission (*p* < 0.01 and *p* = 0.02, respectively), followed by lower peaks and increasing again at day 5 after admission. Similar changes were found in SV for the entire population and within the sub-groups.

Both sexes started with the same CI values at baseline. Shortly after, a sharp increase was evident amongst females, reaching its peak at 48 h after admission (*p* < 0.001), while amongst males a sharp decrease was seen after 24 h (*p* < 0.001), maintained until day 4, in which a further decrease was evident, and a moderate increase appeared on day 5, to levels lower than baseline (*p* < 0.001). Both middle-aged and elderly showed decreases within the first 24 h (*p* < 0.001 in both). Amongst the elderly, a further decrease was evident on day 4, increasing slightly on day 5 (*p* < 0.001). Unlike other age sub-groups, the over 80 years started with a sharp increase in CI during the first 24 h, followed by a sharp decrease and immediately followed with a higher increase by 48 h after admission (*p* < 0.001). This was followed by an unstable decrease over the next two days, and a sharp increase at day 5 (*p* < 0.001). Obese patients had a lower baseline level of CI, further decreasing during the first 24 h, and reaching a nadir at day 5 (*p* < 0.001).

Females had a higher SVR value at baseline as compared with males, and during the 5 days showed a consistent decrease until the end of monitoring (*p* < 0.001). Males started with an increase during the first 24 h (*p* < 0.001) and remained relatively stable until day 5. Middle-aged patients had an increase in the first 24 h (*p* < 0.01) followed by a continuous decrease until day 5, returning to baseline level. Overweight patients showed a continuous decrease during the five days (*p* < 0.01).

## 4. Discussion

In this retrospective multi-center study, we continuously monitored key vitals amongst hospitalized COVID-19 patients using a non-invasive chest-patch sensor. Early after admission, cardiovascular and respiratory parameters deteriorated in parallel, but significant differences in trajectories were found between age, sex, and BMI groups.

While prior studies employed periodic and infrequent biomarkers and echocardiography focusing on cardiac damage, we used continuous monitoring of advanced cardiac parameters using a novel and non-invasive technology, previously showing its capabilities with similar measurements compared to invasive techniques [28]. By using continuous remote patient monitoring combined with health AI we show early cardiovascular changes in COVID-19 patients. Our findings strengthen the notion that frequently measured vital signs, including advanced cardiovascular parameters such as CO, CI, and SVR, might have future implications in the understanding of the progression of COVID-19 in humans, and in particular in high-risk sub-groups [19,20,21,22].

We found that during the first 24 h from admission the changes in the cardiovascular parameters appeared in parallel to the changes in the respiratory parameters, and were more prominent amongst males, patients older than 80 years, and obese patients. Moreover, the decrease found in DBP throughout most of the monitoring period with a concomitant decrease in CO and CI, and increase in SVR, might correlate with prior reports on diastolic dysfunction resulting from COVID-19 damage to the heart [29,30], yet this hypothesis is still to be validated since we did not perform echocardiography. In parallel to the decrease in the cardiovascular parameters, we found an increased average temperature in males, while lower SpO_2_ and increased RR were evident in males, elderly patients, and overweight patients. This could also strengthen previous findings that showed patients with pre-existing respiratory and cardiovascular diseases have an increased risk of severe morbidity and mortality [1,19,31,32,33,34,35].

Additional observations were repetitive patterns of HR, SBP, CO, and CI (Figure 3 and Figure 4). This might be part of a circadian rhythm, yet again, we do not have enough data to substantiate this observation, and it should be further studied.

Significant changes between the studied groups were already apparent during the 2-h baseline period, emphasizing that physiological effects of COVID-19 are different among the various groups beyond the expected naturally occurring differences.

Although statistically significant changes were found in some vitals among the groups, they were slight and are currently not considered to be of clinical significance. However, we think that as advanced monitoring tools will keep developing and being introduced into clinical practice, amplified by advanced AI and machine learning analysis tools, we may find that even these slight changes could have significance and warrant clinical attention, especially when developing early warning score systems in the context of complex patients at high risk of deterioration.

Operationally, the small wearable, wireless RPM device continuously and automatically collected and transferred the data, in real-time, to the medical staff. This reduced the direct contact between medical staff and patients without compromising the medical care provided, an important feature highly required during a pandemic. Moreover, by using this technology with COVID-19 patients we now have an opportunity to define a novel COVID-19 score for the accurate early detection of deterioration. This might also serve as an important component of medical care in the ambulatory and out-of-hospital environments, with early identification of symptomatic and pre-symptomatic infected individuals especially valuable during this period [28,36,37,38].

A limitation of this study is that we did not have the clinical data records and outcomes of these patients. All were admitted to isolation units in a moderate to severe condition, and some were later transferred to COVID-19 dedicated ICUs. We have no information regarding outcomes, administration of supplemental oxygen, vasopressors, and specific therapeutics against COVID-19. However, they all received advanced medical care, and the number of data points of multiple physiological parameters collected was large and frequent, still allowing to have insights of clinical significance. Ongoing studies are now conducted to allow the parallel analysis of collected vitals and clinical data records.

## 5. Conclusions

Frequent monitoring approach using a remote patient monitoring system and advanced bioinformatic tools shows early cardiovascular changes among hospitalized COVID-19 patients. These changes appear in parallel to changes in respiratory parameters, further emphasizing the cardiorespiratory effects of COVID-19 over time, with differential physiological responses noted between sex, BMI, and age groups. This may serve to improve early detection of clinical deterioration of COVID-19 patients, especially important in times of overwhelmed health care systems, helping to reduce direct contact between health care providers and COVID-19 patients without compromising medical care.

## Figures and Tables

**Table 1:** Patient characteristics and average values by sub-groups for the first 2 h of monitoring.

Variable	Sex		Age					BMI			
	Male	Female	Young (18–40 Years)	Middle Age (40–60 Years)	Elderly (60–80 Years)	>80 Years	*p*	Normal <24.9 kg∙min^−2^	Overweight 25.0–29.9 kg∙min^−2^	Obese >30.0 kg∙min^−2^	*p*
*n*	313	179	131	128	171	62		198	171	123	
Sex M: F			84:47	89:39	113:58	27:35		111:87	122:49	80:43	
Monitoring period (hours)	95.4 ± 67.6	83.5 ± 62.6	64.9 ± 43.7	87.8 ± 49.8	101.5 ± 69.3	120.7 ± 97.9		86.6 ± 69.1	98.1 ± 64.1	88.0 ± 63.2	
Age (years)	56 ± 17	60 ±22	31 ± 6 ^b,c,d^	53 ± 5 ^a,c,d^	69 ± 6 ^a,b,d^	87 ± 5 ^a,b,c^	*	53 ± 20 ^&,^^	61 ± 18 ^#^	59 ± 17 ^#^	*
Weight (kg)	83.1 ± 14.5	71.0 ± 15.5	75.7 ± 17.6 ^b^	83.0 ± 14.6 ^a,c,d^	78.8 ± 16.5 ^b^	75.1 ± 11.1 ^b^	*	67.2 ± 10.3 ^&,^^	80.1 ± 9.0 ^#^	95.3 ± 16.3 ^#^	*
BMI (kg∙m^−2^)	27.26 ± 4.49	26.83 ± 5.92 ^$^	25.3 ± 5.33 ^b,c,d^	28.25 ± 4.49 ^a^	27.2 ± 5.36 ^a^	27.63 ± 3.81 ^a^	*	22.74 ± 2.18 ^&,^^	27.41 ± 1.36 ^#^	33.67 ± 4.54 ^#^	*
Body temp. (C)	37.3 ± 0.7	37.2 ± 0.6 ^$^	37.2 ± 0.6 ^d^	37.3 ± 0.7	37.4 ± 0.7 ^d^	37.2 ± 0.7 ^a,c^	*	37.3 ± 0.6	37.3 ± 0.7	37.3 ± 0.7	
SpO_2_ (%)	95.4 ±3.3	95.9 ±2.9 ^$^	96.7 ± 2.4 ^b,c,d^	95.0 ± 3.3 ^a,c^	95.1 ± 3.2 ^a,b,d^	95.7 ± 3.3 ^a,c^	*	96.0 ± 2.9 ^&,^^	95.5 ± 3.0 ^#,^^	95.0 ± 3.6 ^#,&^	*
RR (br∙min^−1^)	17.5 ± 4.2	17.1 ± 3.7	15.7 ± 3.7 ^b,c,d^	18.1 ± 4.1 ^a,d^	18.0 ± 4.0 ^a^	17.1 ± 3.7 ^a,b^	*	17.2 ± 4.0 ^^^	17.0 ± 4.0 ^^^	18.1 ±4.2 ^#,&^	*
HR (beats∙min^−1^)	83 ± 14	81 ± 14 ^$^	83 ± 15 ^d^	84 ± 14 ^d^	82 ±13 ^d^	79 ± 14 ^a,b,c^	*	83 ± 13 ^^^	83 ± 14	81 ± 5 ^#^	
SBP (mmHg)	129 ± 17	128 ± 20	126 ± 15 ^c,d^	128 ± 18 ^d^	130 ± 20	132 ± 23 ^a,b^	*	129 ± 18	128 ± 17	129 ± 20	
DBP (mmHg)	77 ± 13	75 ± 11 ^$^	77 ± 11.7 ^c,d^	78 ± 12 ^d^	75 ± 12 ^c,d^	72 ± 13 ^a,b,c^	*	76 ± 12	76 ± 13	76 ± 12	
CO (l∙min^−1^)	5.9 ± 1.4	5.7± 1.4 ^$^	6.0 ± 1.6 ^d^	5.9 ± 1.3 ^d^	5.8 ± 1.3 ^d^	5.4 ± 1.2 ^a,b,c^	*	5.9 ± 1.5 ^^^	5.9 ± 1.4 ^^^	5.6 ± 1.2 ^#,&^	*
CI (L∙min^−1^∙m^−2^)	3.1 ± 0.9	3.2 ± 0.9	3.3 ± 1.0 ^b,c,d^	3.1 ± 0.8 ^a^	3.2 ± 0.8 ^a,d^	2.9 ± 0.8 ^a,c^	*	3.4 ± 0.9 ^&,^^	3.1 ± 0.8 ^#,^^	2.8 ± 0.8 ^#,&^	*
SVR (dynes∙s^−1^∙cm^−5^)	1311 ± 277	1341 ± 295	1295 ± 291 ^d^	1319 ± 253 ^d^	1313 ± 277 ^d^	1401 ± 336 ^a,b,c^	*	1309 ± 291	1317 ± 284	1350 ± 269	

Values as mean ± SD representing each patient’s entire monitoring period (mean ± SD hrs of monitoring; range: 24–225 h). BMI: body mass index; temp.: temperature; SpO_2_: oxyhemoglobin saturation; RR: respiratory rate; HR: heart rate; SBP: systolic blood pressure; DBP: diastolic blood pressure; CO: cardiac output; CI: cardiac index; SVR: systemic vascular resistance. * Significant interaction (within-group comparison), *p* < 0.05; ^$^ female significantly different than male, *p* < 0.05; ^a,b,c,d^ significantly different than young, middle age, elderly, >80 years within age categories, *p* < 0.05; ^#,&,^^ significantly different than normal, overweight, obese withing BMI categories, *p* < 0.05.

## Data Availability

The datasets generated during and/or analyzed during the current study are available from the corresponding author on reasonable request.
